# Visualization of Global Disease Burden for the Optimization of Patient Management and Treatment

**DOI:** 10.3389/fmed.2017.00086

**Published:** 2017-06-19

**Authors:** Winfried Schlee, Deborah A. Hall, Niklas K. Edvall, Berthold Langguth, Barbara Canlon, Christopher R. Cederroth

**Affiliations:** ^1^Department for Psychiatry and Psychotherapy, University Hospital Regensburg, Regensburg, Germany; ^2^National Institute for Health Research (NIHR) Nottingham Biomedical Research Centre, Nottingham, United Kingdom; ^3^Otology and Hearing Group, Division of Clinical Neuroscience, School of Medicine, University of Nottingham, Nottingham, United Kingdom; ^4^Experimental Audiology, Department of Physiology and Pharmacology, Karolinska Institutet, Stockholm, Sweden

**Keywords:** diagnostic tests, patient management, value-based decision-making, treatment outcome, disease progression, treatment response, subtyping, gender differences

## Abstract

**Background:**

The assessment and treatment of complex disorders is challenged by the multiple domains and instruments used to evaluate clinical outcome. With the large number of assessment tools typically used in complex disorders comes the challenge of obtaining an integrative view of disease status to further evaluate treatment outcome both at the individual level and at the group level. Radar plots appear as an attractive visual tool to display multivariate data on a two-dimensional graphical illustration. Here, we describe the use of radar plots for the visualization of disease characteristics applied in the context of tinnitus, a complex and heterogeneous condition, the treatment of which has shown mixed success.

**Methods:**

Data from two different cohorts, the Swedish Tinnitus Outreach Project (STOP) and the Tinnitus Research Initiative (TRI) database, were used. STOP is a population-based cohort where cross-sectional data from 1,223 non-tinnitus and 933 tinnitus subjects were analyzed. By contrast, the TRI contained data from 571 patients who underwent various treatments and whose Clinical Global Impression (CGI) score was accessible to infer treatment outcome. In the latter, 34,560 permutations were tested to evaluate whether a particular ordering of the instruments could reflect better the treatment outcome measured with the CGI.

**Results:**

Radar plots confirmed that tinnitus subtypes such as occasional and chronic tinnitus from the STOP cohort could be strikingly different, and helped appreciate a gender bias in tinnitus severity. Radar plots with greater surface areas were consistent with greater burden, and enabled a rapid appreciation of the global distress associated with tinnitus in patients categorized according to tinnitus severity. Permutations in the arrangement of instruments allowed to identify a configuration with minimal variance and maximized surface difference between CGI groups from the TRI database, thus affording a means of optimally evaluating the outcomes in individual patients.

**Conclusion:**

We anticipate such a tool to become a starting point for more sophisticated measures in clinical outcomes, applicable not only in the context of tinnitus but also in other complex diseases where the integration of multiple variables is needed for a comprehensive evaluation of treatment response.

## Introduction

Complex health-care conditions can be characterized by a combination of identified and unidentified etiological factors including genetics, environment, and lifestyle (1). Among them are found Alzheimer’s disease, schizophrenia, scleroderma, asthma, Parkinson’s disease, multiple sclerosis, and osteoporosis. Phenotypic heterogeneity in the expression of etiological factors adds complexity to clinical assessment and management and can underlie a mixed response to the same management strategy. Indeed, a new area of research has emerged to characterize complex disorders as profiles that possess a defined set of characteristics (2). Identifying important parameters for patient profiling is a challenging task, yet it is an important step toward being able to provide personalized treatment and would support efforts to develop new treatments. However, in many complex health-care conditions, this has been hard to achieve. Tinnitus is one such example, with a wide range of problems experienced by those who suffer from this condition (3, 4). Tinnitus is defined as the phantom perception of sounds in the absence of any external stimulus. Diagnosis primarily relies on self-report, yet because the impact of tinnitus can be so varied from one patient to another, how it is best treated in individuals is still unclear (5). For example, some patients can complain primarily from sleeping problems (6), or from impaired cognitive function (7, 8) or from communication disabilities (9). Tinnitus is a highly unmet clinical need and there are still no singularly effective therapies that reliably reduce tinnitus percept or its symptoms (10–13). Inter-subject variability in the severity of different tinnitus complaints at diagnostic assessment and at outcome assessment of treatment-related response poses challenges for clinical research. Researchers would benefit from being able to build up an overall picture of the different independent components (or domains) of this complex condition, in order to meaningfully capture key discriminative features between individuals or treatment-related differences.

Based on the tinnitus psychological model of Dauman and Tyler that clearly distinguishes the mechanisms of tinnitus from the reactions to tinnitus (14), the use of multiple measurement instruments has been proposed to address the challenges in quantifying the different aspects of tinnitus (15, 16). However, different laboratories deal with these measures in various ways. One option is the presentation of scores for each measurement instrument. The rationale of this approach is that even if most of these measurements correlate with each other, they assess slightly different aspects of an individual’s tinnitus (17, 18). A further option is the use of statistical methods (namely Principal Components Analysis) to tease apart independent components of the condition in a data-driven way (19). Tyler and colleagues also suggested focusing only on areas that show an impact (20, 21). However, whereas this approach might be useful in clinical practice where a dominant problematic has been found in an individual patient and justifies a primary focus, this should be avoided when performing clinical trials. Indeed, more than a hundred instruments have been used as primary outcome measures in tinnitus clinical trials, which hampers the synthesis of existing evidence (e.g., with meta-analyses) and the delivering of conclusive guidelines for clinical care (22). In order to avoid the random selection of instruments in tinnitus studies and facilitate the comparison and synthesis of clinical data, an approach has been proposed as part of the COMiT initiative (Core Outcome Measures in Tinnitus), which aims to establish an international standard for outcome measurements in clinical trials of tinnitus (23). This approach still uses multiple measurement instruments, but their selection is informed by first identifying in a consensus-manner which tinnitus-related complaints are judged to be the most important ones from the perspective of assessing whether a treatment has been beneficial or not, and then identifying one measurement instrument to assess each relevant complaint (23). This approach thus seeks to tease apart independent components of the condition in a hypothesis-driven way. Independent of which approach is taken, they all require the presentation of multiple measures simultaneously for an individual or group. A graphical visualization approach would facilitate the rapid interpretation and would be more attractive to clinicians and patients than a numerical presentation style. How best to visualize this multiplicity of data and to integrate the complex data patterns into a single clinical interpretation is a challenge that is shared across all approaches described above and that is relevant for many complex disorders.

Any aggregated visualization of data relating to a complex health-care condition should meet a number of requirements to facilitate clinic usage: (i) it should be possible to display measures with different scales (interval, ordinal, etc.), (ii) it should be possible to display individual and group data, with SDs if relevant, (iii) it should be visually appealing, (iv) it should be easy to interpret, and (v) it should be able to represent pre- and post-intervention data.

Here, we introduce a method for the holistic representation of components of health status relevant to a complex multi-attribute condition based on radar plots. Radar plots allow the representation of multivariate data on a two-dimensional graphical illustration and have been suggested as a useful approach for the visualization of multivariate clinical data (24). By selecting tinnitus as a model, we illustrate the usefulness of using radar plots to give a holistic representation of multiple variables used in the assessment of tinnitus. In a first aim, we assess the performance of the radar plots in conveying an ensemble of clinical data from the Swedish Tinnitus Outreach Project (STOP) cohort. In a second aim, we evaluate the performance of the various instruments working together to provide a meaningful overview of treatment outcome using data from the Tinnitus Research Initiative (TRI) database. We propose that this methodology can be applied to any complex clinical disorder where multiple assessment tools are used, for single subjects or group data, cross-sectional or longitudinal data.

## Methods

### Participants

Data are reported from two datasets. The first dataset comes from the STOP recruiting participants with or without tinnitus from the Swedish population^1^ and the second dataset comes from the TRI database.^2^ STOP is a nationwide population cohort with the aims of identifying tinnitus biomarkers. Free-willing registration was done on a website and after participants provided their informed consent, they were invited to fill an online survey (25). The project was approved by the local ethics committee “Regionala etikprövningsnämnden” in Stockholm (#2014/1998-31/4). The database project and server are coordinated and located at the Department of Physiology and Pharmacology of the Karolinska Institutet, Sweden. For this study, a first cross-sectional set of data collected until the 11th of October 2016 included 311 occasional and 328 constant tinnitus subjects. A second cross-sectional set of data was extracted on the 5th of December 2016. That dataset included 1,223 non-tinnitus control subjects and added 294 constant tinnitus subjects. The socio-demographics of the current STOP dataset are presented in Table S1 in Supplementary Material. The second dataset comes from the TRI database (26). and includes 571 individuals who participated in any clinical treatment study at the University Hospital Regensburg, Germany and had consented to reuse of their anonymized data to address new research questions (34.5% female, 65.5% male, age distribution: 17–87 years, mean age ± SD: 52.3 ± 12.2 years). Collection of data for the TRI database was approved by the Ethics Committee of the University of Regensburg, Germany (#08/046).

### Selection of Outcome Domains and Measurement Instruments

A range of health domains were assessed using investigator-administered tests and patient-reported questionnaires. Collectively, they provide an overall clinical impression of global health burden. All domains and associated instruments have been identified from a review of clinical trials of tinnitus treatments in adults (22).

#### STOP Cohort

**Figure 1:**
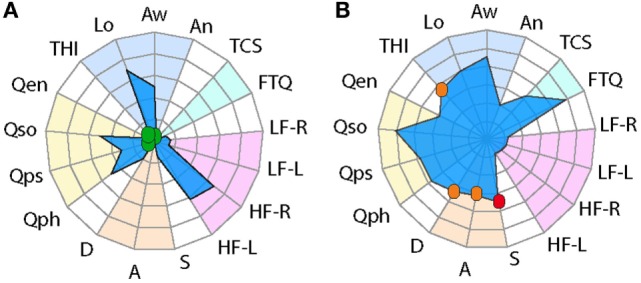
Radar plot profiling for characterizing global health burden in individuals with constant tinnitus. Two tinnitus cases are represented in the radar plots. **(A)** A 72-year-old male subject with high loudness and awareness values displays high-frequency hearing loss but little tinnitus-associated burden. **(B)** A 31-year-old male subject with no hearing loss shows high loudness and awareness scores but with mild/moderate THI scores, moderate depression and anxiety, and severe stress scores. Domains of tinnitus-related burden are grouped in the dark blue region, hearing loss comorbidities in light blue, emotional comorbidities in orange region, and health-related quality of life in yellow. Instruments are labeled as follows: THI, tinnitus-related psychological distress; Lo, tinnitus loudness; Aw, tinnitus awareness; An, tinnitus annoyance; TCS, tinnitus catastrophizing; FTQ, tinnitus fears; LF, low frequency hearing in the left (-L) and right (-R) ears; HF, high-frequency hearing in the left (-L) and right (-R) ears; S, stress; A, anxiety; D, depression scores; Qph, Quality of Life for physical; Qps, psychological, Qso, social; Qen, environment. Color dots illustrate the severity score of those instruments with published severity category boundaries: negligible (green), moderate (orange), and severe (red).

In the STOP cohort, 5 domains of tinnitus and associated comorbidities (psychological distress, tinnitus-related worries and fears, emotional affects, hyperacusis, and quality of life) were measured by 14 separate tinnitus instruments, in which adaptation to Swedish was validated in a previous study (25). In a pilot study, of which two subjects are shown in Figure 1, auditory values were included as an additional measure of hearing loss. The Tinnitus Handicap Inventory (THI) (27, 28) was used to measure tinnitus-related psychological distress. Participants rated each of the 25 items on a categorical 3-point scale (“yes” = 4/“sometimes” = 2/“no” = 0). The mean global score reflects the sum of all responses with a maximum score of 100 indicating the greatest impact on everyday function. For the purposes of analysis, the THI global score was used since it is considered a unidimensional measure (29). The THI cutoffs were defined previously (30) and are split in five different categories from slight (0–16), mild (18–36), moderate (38–56), severe (58–76), and catastrophic (78–100). For sake of clarity and the rapid interpretation of severity, we combined light and moderate together as well as severe and catastrophic. In the radar plots, three colors helped classifying severity: green (negligible), orange (light/moderate), and red (severe/catastrophic). Three Numerical Rating Scales were taken from the Tinnitus Sample Case History Questionnaire (26). One measured tinnitus loudness (Lo): “Describe the loudness of your tinnitus using a scale from 1 (very faint) to 100 (very loud).” One measured tinnitus awareness (Aw): “What percent of your total awake time, over the last month, have you been aware of your tinnitus?” The third measured tinnitus annoyance (An): “What percent of your total awake time, over the last month, have you been annoyed, distressed, or irritated of your tinnitus?”

The Fear of Tinnitus Questionnaire measures the worries and fears of patients experiencing tinnitus [FTQ; (31)]. There are 17 items that are rated on a true or false scale. A greater score indicates more extreme fear. Catastrophic cognitive misinterpretations of tinnitus sounds were measured by the Tinnitus Catastrophizing Scale [TCS; (31)]. This is an adapted version of the Pain Catastrophizing Scale in which the word “pain” was substituted by the word “tinnitus” (32). There are 13 items, in which participants indicated the extent to which each statement applies to them using a 5-point scale (“always” = 4 to “not at all” = 0). A greater score indicates more extreme perceptions. TCS has a unidimensional structure and the global score was used (31).

Five important comorbidities are anxiety, depression, stress, hearing loss, and hyperacusis. Measurement instruments for each domain are described as follows: anxiety and depression were measured by the Hospital Anxiety and Depression Scale [HADS; (33)]. HADS comprises 7 items on anxiety (A) and 7 items on depression (D), with each item scored from 0 to 3. Higher scores indicate greater severity, with the maximum score being 21. The HADS cutoffs were defined previously by Zigmond and Snaith (33) and are split in three different categories from normal (0–7, shown in green), borderline (8–10, shown in orange), and abnormal (11–21, shown in red). The Perceived Stress Questionnaire assesses chronic and acute relationships with stressful events and activities [PSQ-30; (34)]. Thirty items are answered using a 4-point scale, from “almost always” = 4 to “almost never” = 1. The sum of the answers is substracted by 30 and the resulting value is divided by 90, yielding a score between 0.0 and 1.0. Higher scores indicate more severe perceived stress. There is no consensus about the factor structure of the PSQ-30 (34, 35) and so here we used the global score, not any subscale scores. The PSQ-30 cutoffs were defined previously by Levenstein et al. (34, 35) and are split in three different categories from low stress level (<0.34, shown in green), moderate stress level (0.34–0.46, shown in orange), and high stress level (>0.46, shown in red).

Hyperacusis is defined as sound intensities that others would find normal, but are experienced as intolerably loud by affected individuals. This marked intolerance to everyday environmental sounds happens even at moderate levels, in spite of quite often normal hearing thresholds. We measured the condition using the Hyperacusis Questionnaire [HQ; (36)]. The second part of the questionnaire comprises 14 negatively worded items, which are rated on a 4-point scale (“yes, a lot” = 3 to “no” = 0). The total provides the measure of hypersensitivity to sound with higher scores indicating greater sensitivity. The maximum global score is 42 and a global score greater than 28 indicates clinically significant hyperacusis (shown in red), while a global score equal to or less than 28 indicates a negligible problem (shown in green). Again there is no consensus about the factor structure of the HQ (36, 37) and so here we used the global score, not any subscale scores.

For the two tinnitus cases presented in Figure 1, hearing loss was reported separately for both low and high frequencies. Hearing was assessed by fixed frequency Bekesy audiometry using a Madsen Astera 2 audiometer and Sennheiser HDA 200 headphone at standard and high audiometric frequencies. Hearing thresholds reported in dB HL (hearing level) were averaged from 0.125 to 6 kHz for lower frequencies. High-frequency thresholds were averaged for frequencies between 8 and 16 kHz. Thresholds were reported separately for left and right ears.

Four domains of health-related quality of life formed the last set of measures [physical health (Qph), psychological (Qps), social relationships (Qso), and environment (Qen)]. We used the WHOQOL-BREF which is a 26-item questionnaire providing a broad reliable measurement with four validated subscale scores (38). Each item has a range of 1–5 and the four domain scores are scaled in a positive direction with higher scores indicating a more positive quality of life. The items on the quality of life must be reversed before scoring.

#### TRI Database

For the assessment of tinnitus within cases from the TRI database, the WHOQoL-BREF, THI, and five numeric rating scales (0–10) were used (26). Numeric rating scales refer to tinnitus loudness (“How STRONG or LOUD is your tinnitus at present?” Tlou), tinnitus annoyance (“How ANNOYING is your tinnitus at present?” Tann), ability to ignore tinnitus (“How easy is it for you to IGNORE your tinnitus at present?” Tign), tinnitus unpleasantness (“How UNPLEASANT is your tinnitus at present?” Tunp), and the uncomfortable aspect of tinnitus (“How UNCOMFORTABLE is your tinnitus at present, if everything around you is quiet?” Tunc). For assessing perceived treatment-related change, an additional measure was the Clinical Global Impression (CGI) scale (39). This is a single question asked at the end of treatment and requires the patient to give an overall rating of his/her current state compared to the pretreatment baseline. The response is scored on a 7-point scale (“very much better” = 1; “much better” = 2; “minimally better” = 3; “no change” = 4; “minimally worse” = 5; “much worse” = 6; “very much worse” = 7).

### Designing the Visualization

A radar plot displays multivariate data in two dimensions. The radar plot comprises a sequence of equi-angular spokes (radii), with each spoke representing one of the measures. The data length of a spoke is proportional to the magnitude of the measurement score, from minimum at the center to maximum at the circumference. A line can then be drawn connecting the data values for each spoke. This gives the plot a star-like appearance and a quantifiable surface area. A patient with high scores across multiple measures is represented by a large surface area (high burden) and conversely a patient with low scores is represented by a small surface area. Evaluation of overall burden takes into account the shape of the radar plot and the size of the plot to address in which domains there are greatest burden experienced. When plots contained two average datasets such as for comparisons of gender or treatment, colors such as blue for men and pink for women, or yellow for pretreatment and orange for posttreatment, were used. Several forms of color coding can be used to facilitate the visualization of the data. Observers do not require any specialized knowledge of the measurement instruments to make an overall judgment about the patient profile. Its interpretation is intuitive. Nevertheless, to support the interpretation of those instruments with published severity category boundaries, we represented negligible problem in green, mild/moderate problems in orange, and severe problems in red. This novel feature of the graphical representation helps the observer to rapidly determine individual burden.

For the STOP dataset, five domains were considered including (i) tinnitus severity (assessed by four instruments, blue background), (ii) tinnitus-related fears (assessed with two questionnaires, light blue background), (iii) hyperacusis (measured with one questionnaire, shown in purple), (iv) emotional comorbidities (assessed by two instruments, orange background), and (v) health-related quality of life (assessed with one questionnaire, yellow background). All scores were adjusted to the same minimum–maximum scale of 0–100. For the TCS, FTQ, and HADS, this was done calculating the percentage of the maximum score (total score/maximum score × 100). The PSQ with a maximum score between 0 and 1 was multiplied by 100. The THI and Numerical rating scales all result in a score between 0 and 100 and were left unmodified. Since the WHOQoL-BREF has higher scores with better life quality, we inversed the scale (100 total score) so that the interpretation of the 0–100 scale was consistent across all measurement instruments. The score from the WHOQoL-BREF was translated into a 0–100 score using the method provided in the WHOQoL user manual using the formula: TRANSFORMED SCORE = (SCORE-4) × (100/16). A value of 100 corresponds to greater severity of negative symptoms. Average hearing threshold values were obtained only in the individual examples and were left unmodified as the range from 0 dB HL (normal hearing) to >90 dB HL (profound) covers the full range of expected hearing loss (40). For the TRI dataset, a smaller number of domains were available between baseline and follow-up and thus consisted of (i) health-related quality of life (measured with the WHOQoL-BREF), (ii) the tinnitus-related psychological distress (assessed with the THI), and (iii) the individual aspects of tinnitus assessed by the five numeric rating scales.

### Statistical Methods

95% confidence intervals were obtained according to the formula:Z*std/sqrt(*n*). Group differences were tested by a two-way ANOVA, and multiple comparison tests were mentioned in the legends (Prism version 4.0, GraphPad software). Differences were considered significant if *p* < 0.05.

## Results

During a pilot study from the STOP, two tinnitus cases were identified that displayed strikingly different tinnitus-associated burden (Figure 1). While both cases displayed relatively high tinnitus loudness and awareness, their radar plot profile indicated a different health burden. Since the THI, the HADS, and the PSQ-30 contain cutoffs for different degrees of severity, these were marked with a color label to grade negligible (green), moderate (orange), or severe (red) scores. A blue surface was used to specify the male gender. A 72-year-old male has a comorbid high-frequency hearing loss, but low scores on tinnitus-related psychological distress (THI), negligible stress (S), anxiety (A), or depression (D) and good quality of life (Qph, Qps, Qso, and Qen) (Figure 1A). By contrast, another male, 31 years old, shows good hearing, mild/moderate tinnitus-related psychological distress (THI), moderate stress (S) and anxiety (A), severe depression (D), and poorer quality of life (Qph, Qps, Qso, and Qen) (Figure 1B). These two examples capture distinct tinnitus-associated burden across individuals. Based on these findings, we utilized the radar plot as a visualization instrument for assessing cross-sectional data in the STOP cohort.

### Cross-sectional Questionnaire-Based Profiling in the STOP Cohort

**Figure 2:**
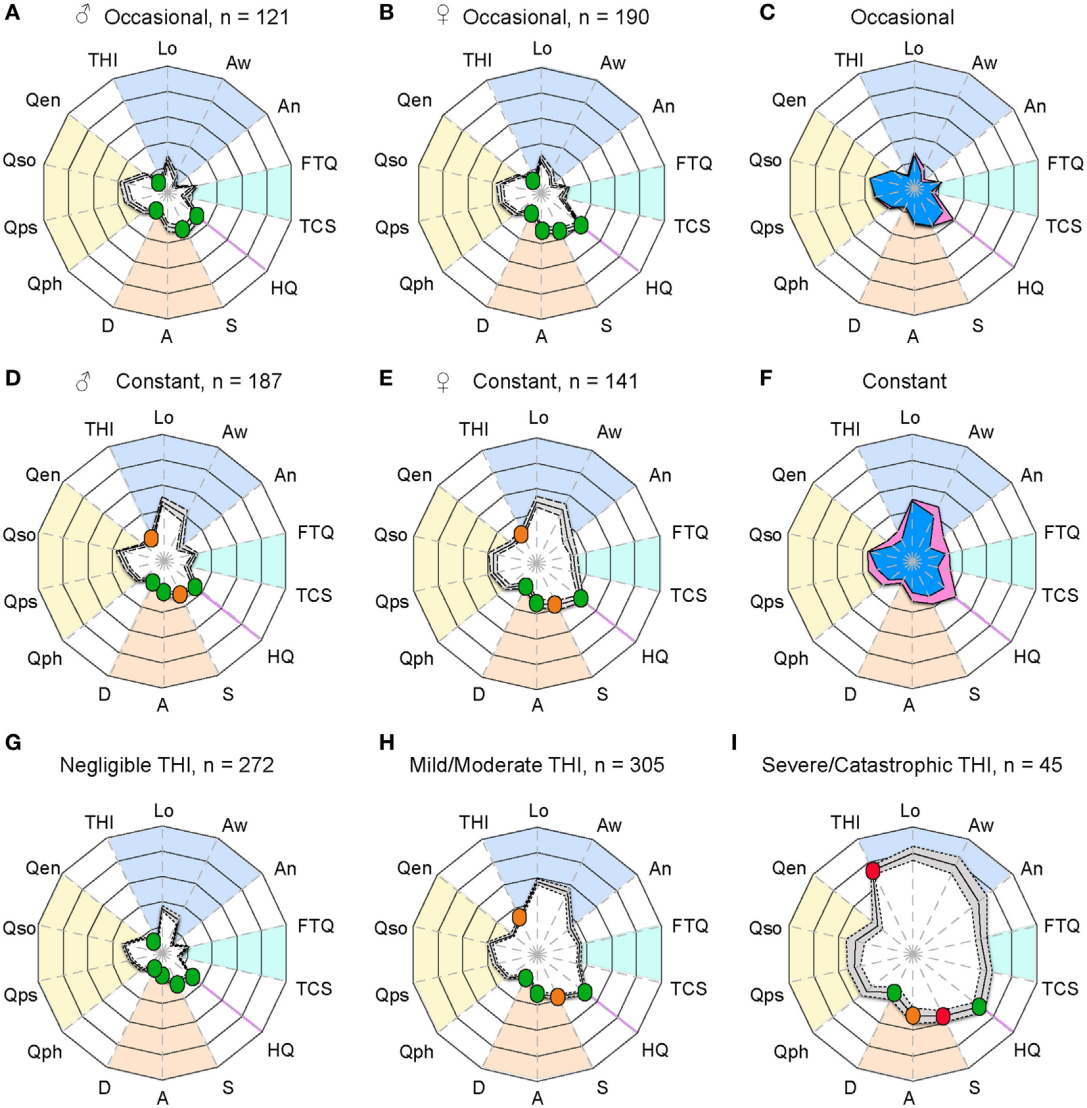
Radar plot profiling that characterizes greater burden in women with tinnitus than in men. Radar plots illustrating the evaluation of global changes in tinnitus burden according to occasional tinnitus for men **(A)** and women **(B)** and constant tinnitus for men **(D)** and women **(E)**. In all four plots, a solid line shows the average, with the 95% confidence intervals represented by the dashed lines. Average tinnitus-associated burden, for men (blue) and women (pink) is shown for occasional **(C)** and constant **(F)** tinnitus. Measures from **(G)** negligible, **(H)** mild/moderate, and **(I)** severe/catastrophic THI groups from the STOP database. The blue background gathers the tinnitus domain assessed with the THI and several numerical rating scales, the light blue represents the tinnitus-associated fears, the hyperacusis domain is marked in purple, emotional affects are in orange, and the yellow background represents the quality of life domain evaluated with the WHOQoL-BREF. The continuous line shows the average scores, and the dashed lines illustrate the 95% confidence intervals. Instruments are labeled as follows: THI, tinnitus-related psychological distress; Lo, tinnitus loudness; Aw, tinnitus awareness; An, tinnitus annoyance; TCS, tinnitus catastrophizing; FTQ, tinnitus fears; HQ, hyperacusis questionnaire; S, stress; A, anxiety; D, depression scores; quality of life for physical (Qph), psychological (Qps), social (Qso), and environment (Qen). Color dots illustrate the severity score of those instruments with published severity category boundaries: negligible (green), moderate (orange), and severe (red).

The two examples provided in Figure 1 are clearly distinguishable from one another, but how do tinnitus profiles vary in a larger sample? To investigate this question, we analyzed information based on the abovementioned measurement instruments gathered from 639 participants who reported occasional or constant tinnitus in the ongoing STOP study. Since the auditory assessment is still ongoing, these values were not included here. We compared the profiles between occasional and constant tinnitus and took the opportunity to evaluate the differences between men and women (Figure 2; Table 1). The tinnitus-associated burden denoted by the surface area of each plot appeared greater in constant tinnitus, than in occasional tinnitus (Figure 2). Although most scores indicated negligible symptoms in the occasional tinnitus group, scores for the THI and the anxiety subscale of the HADS were mild/moderate in constant tinnitus (Table 1). When assessing gender differences for intermittent tinnitus, the overall burden appeared very similar between men and women, with the exception of hyperacusis whereby the plots highlighted a small difference in scores [♀: HQ = 16.60 (15.38–17.82), and ♂: HQ = 12.32 (10.88–13.75), Figure 2C]. However, while this difference was statistically significant (*p* < 0.0001), both gender subgroups were below the grading of a clinically meaningful hyperacusis (cutoff = 28). This difference between men and women can be appreciated in the radar plots where an extension of the HQ radii can be seen in women (Figure 2C), indicating that the radar plots allow to visually capture subtle changes. For constant tinnitus, many of the tinnitus domains had greater scores in women than in men, including awareness, annoyance, and catastrophic cognitive misinterpretations (Figure 2F). Comorbidities were also slightly higher, including hyperacusis and anxiety. Psychological distress (THI) and stress (HADS) were the only instruments showing a shift from the negligible toward the moderate category. Analysis of the data using a two-way ANOVA confirmed that genders differed significantly [*F*_(1, 8,715)_ = 193.4; *p* < 0.0001, Table 1]. Again, the visualization of the radar plots merged for men and women enable to observe a greater surface area for women.

**Table 1:** Raw scores from STOP participants with occasional or constant tinnitus according to gender.

Occasional tinnitus			

	♂ (*N* = 121)	♀ (*N* = 190)	Two-way ANOVA (*p* value)
	Average ± CI	Average ± CI	
THI	10.35 (8.07–12.63)	11.51 (9.76–13.26)	<0.9999
Lo	25.87 (22.43–29.3)	28.56 (25.62–31.49)	0.228
Aw	13.66 (10.76–16.56)	17.97 (14.8–21.13)	0.6937
An	6.97 (5–8.94)	10.65 (8.09–13.21)	0.8108
FTQ	3.76 (3.37–4.16)	3.61 (3.31–3.92)	>0.9999
TCS	7.02 (5.7–8.33)	8.61 (7.34–9.87)	>0.9999
HQ	12.32 (10.88–13.75)	16.6 (15.38–17.82)	***<0.0001
S	0.33 (0.3–0.36)	0.33 (0.31–0.36)	>0.9999
A	5.7 (5.01–6.39)	5.92 (5.33–6.51)	>0.9999
D	3.32 (2.77–3.86)	3.24 (2.75–3.73)	>0.9999
Qph	16.25 (15.83–16.67)	15.93 (15.53–16.32)	0.4321
Qps	14.98 (14.47–15.5)	14.84 (14.45–15.23)	0.9863
Qso	14.31 (13.77–14.85)	14.6 (14.18–15.02)	>0.9999
Qen	16.45 (16.06–16.84)	16.4 (16.09–16.7)	>0.9999

**Constant tinnitus**			

	**♂ (*N* = 187)**	**♀ (*N* = 141)**	**Two-way ANOVA (*p* value)**
	**Average ± CI**	**Average ± CI**	

THI	18.73 (16.47–20.99)	26.29 (23.19–29.38)	***0.0001
Lo	47.24 (43.78–50.69)	49.16 (45.05–53.26)	0.0031
Aw	40.23 (35.52–44.94)	47.84 (42.67–53.01)	***<0.0001
An	17.69 (14.48–20.9)	31.32 (26.73–35.9)	***<0.0001
FTQ	4.56 (4.19–4.93)	5.1 (4.66–5.54)	0.7674
TCS	10.55 (9.27–11.83)	16.24 (14.54–17.94)	***<0.0001
HQ	13.75 (12.55–14.96)	18.89 (17.35–20.44)	***<0.0001
S	0.3 (0.28–0.33)	0.37 (0.33–0.4)	**0.0046
A	5.15 (4.61–5.69)	6.54 (5.78–7.29)	***0.0002
D	3.24 (2.8–3.67)	3.94 (3.28–4.61)	0.6755
Qph	16.22 (15.88–16.57)	15.2 (14.71–15.69)	*0.0184
Qps	15.57 (15.23–15.91)	14.44 (13.95–14.92)	**0.0066
Qso	14.49 (14.08–14.89)	14.32 (13.76–14.89)	>0.9999
Qen	16.83 (16.55–17.12)	15.94 (15.54–16.35)	0.1123

*See Figure 1 for a description of the abbreviations for each measure. Average values and 95% confidence intervals are shown. Pairwise comparisons using Bonferroni tests are reported*.

*p < 0.05.

**p < 0.001.

***p < 0.001.

Although the radar plots allowed to distinguish contrasting tinnitus-associated burden in constant tinnitus versus occasional as well as subtle differences between genders in both subgroups, we sought to determine whether the radar plots could support the interpretation of those instruments with published severity category boundaries. To do so, an additional set of questionnaire data was collected from 294 subjects with constant tinnitus and added to the previous dataset of 328 for a total of 622 subjects with constant tinnitus. These were classified according to three grades of the THI from slight (range: 0–16, *n* = 272), mild and moderate (range: 18–56, *n* = 305), and severe to catastrophic (range: 58–100, *n* = 45). The HQ, HADS, PSQ-30, and WHOQoL-BREF data from the slight group did not differ from that of non-tinnitus control subjects (*n* = 1,845, Table 2). Consistently, the radar plots showed no clinically meaningful scores for the slight THI group (Figure 2G); however, the mild/moderate THI groups displayed an increased in tinnitus-associated burden with stress scores being *moderate* (Figure 2H; Table 2), whereas the severe/catastrophic THI group showed much larger surface of the radar plots with *severe* stress and *moderate* anxiety levels (Figure 2I; Table 2). Hyperacusis scores increased significantly from negligible to mild/moderate and from mild/moderate to severe/catastrophic tinnitus groups, but remained below clinically meaningful hyperacusis values (Table 2). Overall, the radar plots facilitated the appreciation of greater tinnitus-associated burden in cross-sectional data from the STOP cohort and suggest that this visualization tool may help in evaluating global tinnitus burden.

**Table 2:** Raw scores from STOP participants according to different THI subgroups.

		THI severity subgroups		
Instruments	Controls (*N* = 1,223)	Negligible (*N* = 272)	Mild/moderate (*N* = 305)	Severe/catastrophic (*N* = 45)
THI		8.6 (8–9.2)	31.1 (29.9–32.2)^a^	72.4 (68.9–75.9)^a,b^
VAS Lo		34.9 (32.7–37.2)	56.8 (54.3–59.3)^a^	79.1 (73.9–84.3)^a,b^
VAS Aw		28.8 (25.5–32)	54.6 (51–58.2)^a^	77.3 (70.1–84.5)^a,b^
VAS An		9.8 (8–11.5)	33 (30.1–35.9)^a^	66.2 (58.5–73.9)^a,b^
FTQ		3.6 (3.4–3.8)	5.8 (5.5–6.1)	9.3 (8.6–10.1)^c^
TCS		6.8 (6.2–7.4)	17.4 (16.5–18.3)^a^	30.9 (28.5–33.2)^a,b^
HQ	11.4 (11–11.8)	12 (11.1–12.9)	20.1 (19.2–21)^a^	27.9 (25.5–30.3)^a,b^
S	0.3 (0.3–0.3)	0.3 (0.2–0.3)	0.4 (0.4–0.4)	0.5 (0.5–0.6)
A	4.8 (4.6–5)	4.1 (3.7–4.5)	6.5 (6.1–7)^d^	10.2 (8.9–11.5)^c^
D	2.6 (2.4–2.7)	2.5 (2.2–2.9)	4 (3.6–4.4)	7.1 (5.9–8.4)
Qph	16.9 (16.8–17)	16.7 (16.5–17)	15.3 (15–15.6)	12.5 (11.7–13.4)
Qps	15.7 (15.6–15.9)	16 (15.7–16.2)	14.6 (14.3–14.9)	12.4 (11.6–13.3)
Qso	14.9 (14.8–15.1)	15.1 (14.8–15.4)	13.9 (13.5–14.2)	12.6 (11.4–13.7)
Qen	17 (16.9–17.1)	17 (16.8–17.3)	16.1 (15.8–16.4)	14.3 (13.5–15.1)

*See Figure 1 for a description of the abbreviations for each measure. Average values and 95% confidence intervals are shown. Multiple comparisons from Holm–Sidak’s tests are reported in superscripts, where different letters represent a statistically significant difference. ^a^p < 0.001 compared with negligible THI, ^b^p < 0.001 compared with mild/moderate THI, ^c^p < 0.01 compared with negligible THI, ^d^p < 0.05 compared with negligible THI. Controls did not differ from negligible THI scores*.

### Evaluation of Treatment Outcome with the TRI Database

We reasoned that these plots could also be informative with respect to treatment-related response. To investigate this question, we analyzed information from the TRI database that contains information from baseline and posttreatment measures collected from 574 individuals based on three sets of questions (THI, WHOQoL-BREF, and tinnitus-related numeric rating scales). The mean age of the group was 53.3 years (SD 12.2) with an average tinnitus duration of 8.4 years (SD 8.87). Women made up 36.7% of the group. These patients were classified into different groups based on the CGI rating (Table 3). Only three patients rated their posttreatment state as “very much worse” and as a consequence this category was excluded from analysis (*N* = 571). We note that the size of the “very much better” and “much worse” groups were also rather small (*n* = 18 and *n* = 24, respectively).

**Table 3:** Categorization of the 571 participants from the Tinnitus Research Initiative database according to their posttreatment CGI rating.

CGI rating	*N*
Very much better	18
Much better	66
Minimally better	131
No change	255
Minimally worse	77
Much worse	24

We hypothesized that the surface area of the radar plots could be used to evaluate and quantify treatment efficacy. However, for a more sensitive measure of treatment outcome, one has to consider the order of the measures around the circumference since the surface area could be influenced depending on which instruments are situated next to each other. It is thus conceivable that a specific arrangement of the axis makes the radar plot tool more sensitive for displaying clinical changes than other possible arrangements. With the restriction that instruments forming part of the same domain should be displayed in vicinity to each other (e.g., WHOQoL-BREF), we simulated the full set of possible permutations, which allowed a number of 34,560 different variants of the radar plot. For each of these variants, the following steps were repeated:

– Plots for all tinnitus patients were created and the surface area calculated.
– Patients were grouped to the CGI categories (see Table 3), the average surface area was calculated for each category to compute the mean distance between groups.
– Patients were grouped to the CGI categories (see Table 3) and the variance of the surface area within each category calculated.

**Figure 3:**
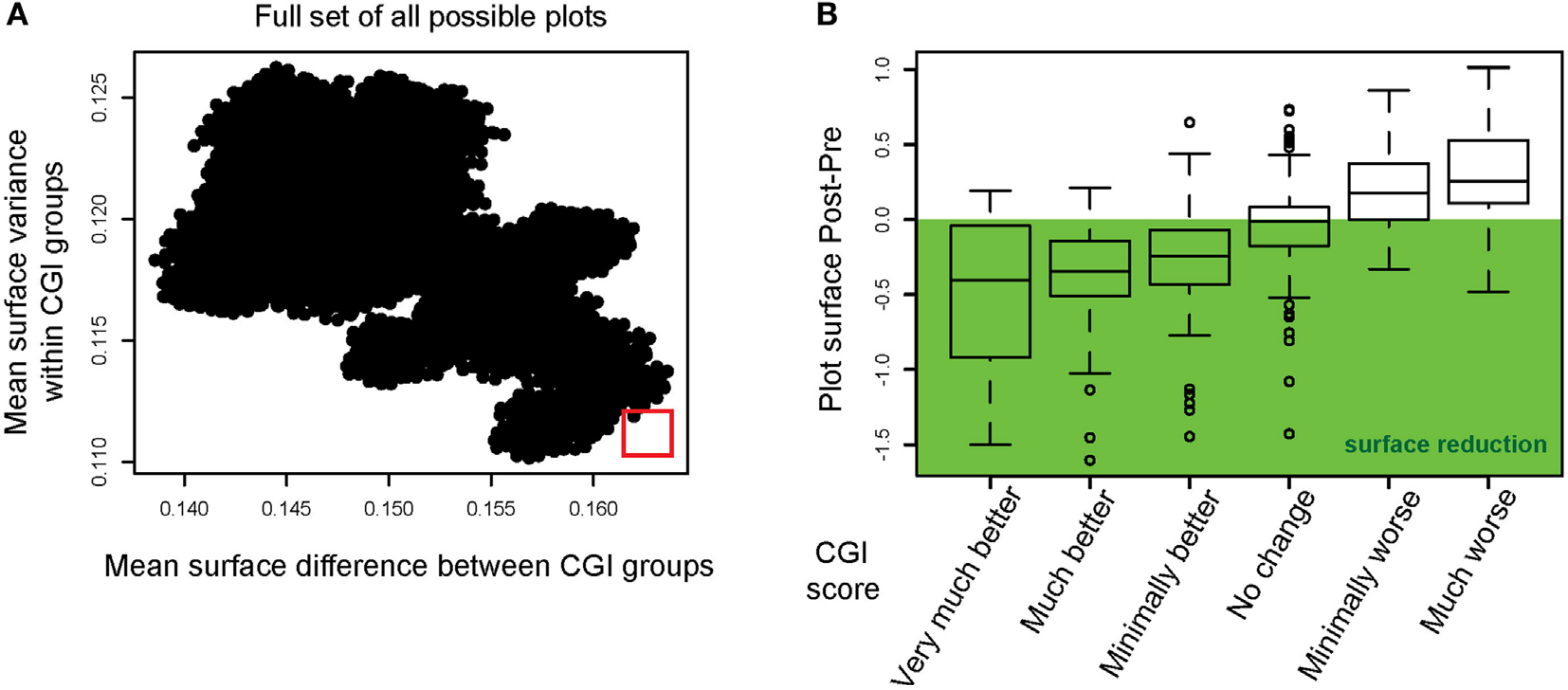
Instrument permutations and alignment of the radar plot with improvements in Clinical Global Impression. **(A)** The full set of all possible radar plots with different surface outlines (i.e., different organization of axes) was simulated. The mean surface difference between CGI groups was calculated for all radar plots (abscissa). The mean surface variance within CGI groups was calculated and plotted on the ordinate. The red square highlights the radar plot outline optimized for maximum difference *between* CGI categories and minimum variance *within* CGI categories. **(B)** Radar plot surface differences (post-intervention minus pre-intervention) are displayed for the optimal outline. The mean surface difference is shown for each CGI category.

The results of these calculations are displayed in Figure 3A with the mean surface difference between CGI categories plotted against the mean surface variance within categories. The goal of this step was to select a figure outline that minimized the surface variance within CGI categories and maximized the mean surface difference between CGI categories (marked with a red square). This radar plot configuration appeared as optimal to the set of instruments used within the TRI database. In Figure 3B, we show the mean difference in radar plot surface area pre- and post-intervention for each CGI category using the selected radar plot configuration. The CGI category “no change” showed on average no change in the plot’s surface, while CGI categories with an improvement show a reduction, while CGI categories with a worsening of the patients’ symptoms are characterized by an increase of the plot’s surface.

**Figure 4:**
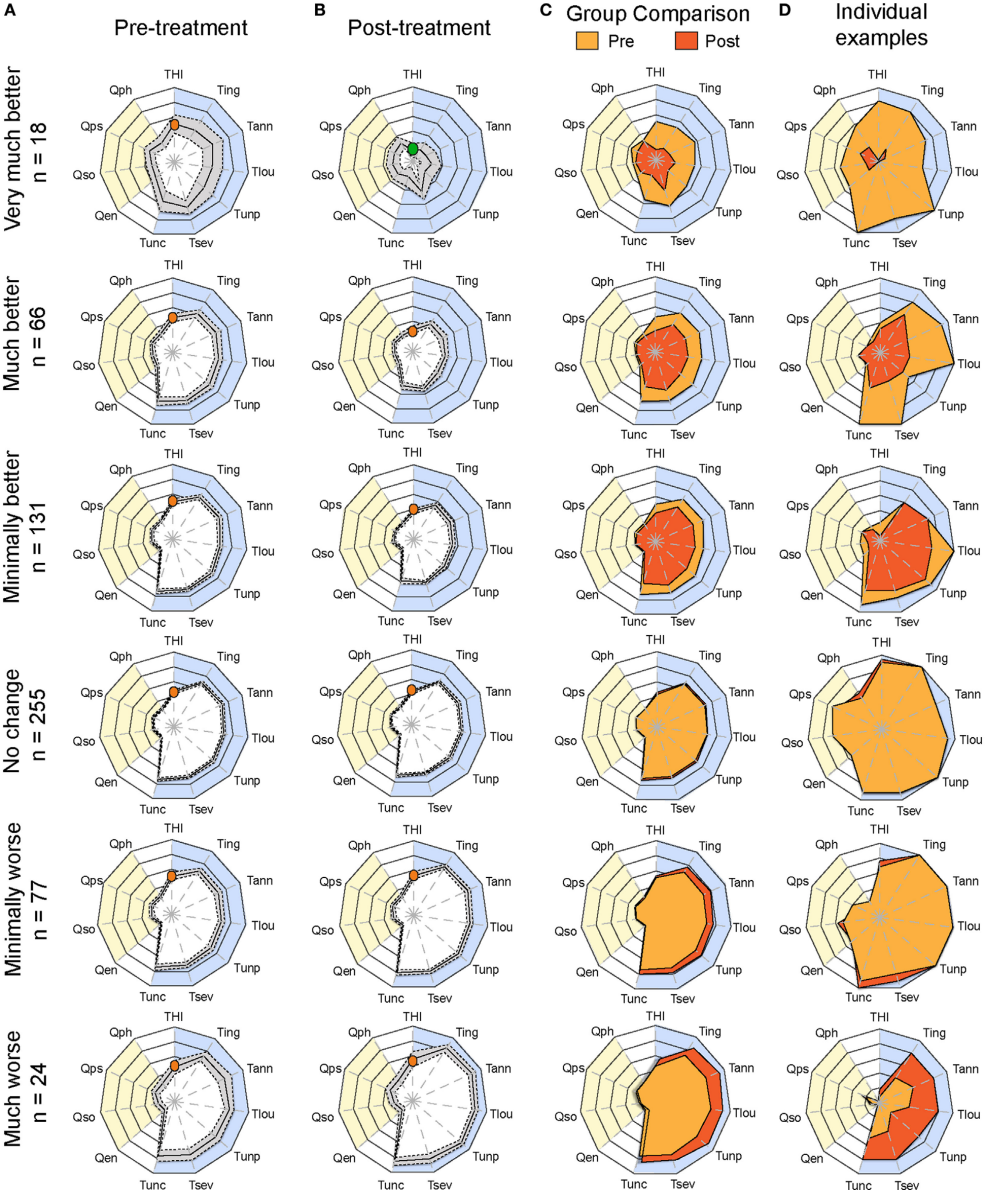
Plots of average pretreatment and posttreatment scores based on CGI ratings. Radar plots illustrating the evaluation of global changes in tinnitus burden according to measures from **(A)** baseline (pretreatment) and **(B)** follow-up (posttreatment) during the evaluation of various treatments within the TRI database. Patients were grouped according to Clinical Global Impression scores and their number is shown on the left. The yellow background represents the quality of life domain evaluated with the WHOQoL-BREF and the blue background gathers the tinnitus domain assessed with the THI and several numerical rating scales. The continuous line in the pretreatment and posttreatment shows the average scores, and the dashed lines illustrate the 95% confidence intervals. **(C)** Pretreatment (yellow) and posttreatment (orange) average values are presented on the same plot. The orange dot in the THI scale marks the moderate scores of the group for this instrument, whereas the green shows the progression of the group to negligible THI. Note the reduction of tinnitus-associated burden in the “very much better” group. **(D)** Example of individuals within each CGI category to illustrate changes before and after treatment for the same patient. Instruments are labeled as follows: THI, Tinnitus Handicap Inventory; Tign, Tinnitus Numeric Rating Scale (Ignore); Tann, Tinnitus Numeric Rating Scale (Annoyance); Tlou, Tinnitus Numeric Rating Scale (Loudness); Tunp, Tinnitus Numeric Rating Scale (Unpleasant); Tsev, Tinnitus Numeric Rating Scale (Severity); Tunc, Tinnitus Numeric Rating Scale (Uncomfortable); Qen, WHO Quality of Life (environment); Qso, WHO Quality of Life (social relationships); Qps, WHO Quality of Life (psychological); Qph, WHO Quality of Life (physical health). Statistical analyses comparing **(A,B)** are available in Table S2 in Supplementary Material.

Having determined the optimal configuration of the radar plot to visualize and score treatment-related changes in CGI groups, we plotted the average radar plots of each of the six CGI categories pretreatment and posttreatment (Figures 4A,B). All CGI groups in the improvement categories displayed significant reduction in surface [two-way ANOVA; very much better: *F*_(1, 374)_ = 77.47, *p* < 0.0001; much better: *F*_(1, 1,430)_ = 155.3, *p* < 0.0001, minimally better: *F*_(1, 2,860)_ = 162.3, *p* < 0.0001]. With increasing power in the “much better” and “minimally better” groups, significant improvement was detected for all instruments, with the exception of the quality of life domain (Table S2 in Supplementary Material). Conversely, in the “minimally worse” group, significant changes were observed [two-way ANOVA; *F*_(1, 506)_ = 28.88, *p* < 0.0001] in particular for the numeric rating scales of loudness, annoyance, ability to ignore, and unpleasantness, while the THI did not detect any worsening (*p* > 0.9999, Table S2 in Supplementary Material). Merging the pretreatment and posttreatment averages on the same radar plot helped appreciating these changes (Figure 4C). Figure 4D illustrates examples of individuals within each CGI category. As shown in these examples, changes over time can be displayed and appreciated by comparing pretreatment and posttreatment plots.

## Discussion

This article describes an innovative visualization method for displaying patient profiles, both to aid clinical assessment and evaluate the effects of treatment-related change, here applied in the context of tinnitus. The method can be adapted to individual patients (e.g., Figures 1 and 4D) as well as on a group level (e.g., Figures 2 and 4A–C). The present method uses data from a multi-dimensional set of relevant measurement instruments and integrates them into a radar plot. Both total scores on domain-specific questionnaires (e.g., the HQ, HADS, and WHOQoL-BREF) and single-item numeric rating scales as well as a psychoacoustic tests can be incorporated to provide an overview in a single plot. The data representation facilitates the visualization of both individual and group data and gives an understandable representation of burden status in a manner that is accessible to a range of observers. Moreover, the representation is sensitive to changes over time and enables the detection of clinically significant improvements in treatment outcome. Different coloring methods can be used such as in Figures 1, 2 and 4 to highlight specific aspects of the data. Usability studies involving practitioners will be needed to decide which coloring method should be used to optimally display the clinical data.

Saary first proposed radar plots for their use of health-care data (24). Funabiki et al. applied this methodology in the context of the assessment of pervasive developmental disorder and attention-deficit/hyperactivity disorder (41). Similarly, Pierzycki et al. used radar plots to visualize factor structure and test–retest reliability of multiple baseline test scores in the context of tinnitus (19). However, all these studies did not apply this methodology in the context of the evaluation of treatment outcome. To the best of our knowledge, this is the first attempt in the medical area to design a comprehensive visualization tool of integrated measures over time. Here, we show the sensitivity of this tool for the assessment of tinnitus patients at their initial consultation at the clinic and for monitoring their progress under a specific treatment. The methodology presented here suggests that it could be applied for monitoring patients throughout their therapeutic intervention. In our examples, a decrease in the radar plot surface could be interpreted as a clinical improvement, while an increase of the surface could be interpreted as a worsening of clinical symptoms (see Figure 4D for individual examples). An important aspect of this use case is the graphical implementation of the minimal clinically important difference (MCID). Meaningful clinical differences need to be highlighted in order to dissociate them from differences that are not of clinical relevance. Further research will be needed to define and implement the MCID in the radar plots.

We also propose that this approach could be applied to other complex disorders where multiple measures are incorporated. We are not stating that the total surface of the area, which can be interpreted as a *global burden* score, can replace the use of a single primary outcome instrument. Rather such visualization tool can be used to appreciate global changes associated with the status of a patient and his responsiveness to a treatment. Although useful for group comparisons, this tool appears to have particular utility in individual patient management strategies. Additionally, specific color-coding methods can be implemented in order to highlight specific aspects of the plot and mark meaningful clinical differences on instruments that have known thresholds of severity. Further work will be needed to develop and standardize a radar plot that is designed to optimally display the clinical status of the tinnitus patients.

An important leverage is the order of the axis used to create the radar plot. As we showed, the selection of the axis order has a strong impact on the surface and surface difference between two time points. Whether instruments need to be grouped according to broad fields such as those used here (e.g., tinnitus, quality of life, emotional burden, and auditory profiles) or instead according to instrument subscales (sleep, cognitive, intrusiveness, relaxation, sense of control, and auditory performance) remains to be established. Furthermore, it is likely that varying the number of data points on the radar plots may also affect the precision and the sensitivity of the tool. The STOP data contain a greater number of instruments than in the TRI data; however, it does not contain treatment outcome data. The TRI data utilized in the present work are limited to (i) the THI, for which validity and reliability has been questioned, (ii) visual analog scales, for which precision is also subject to debate, and (iii) the WHOQoL, which usefulness in the evaluation of treatment outcome is unclear. As a consequence, to evaluate the influence of the number of data points on the precision of the radar plots, studies will have to be performed using a larger set of instruments. This will allow a greater flexibility in evaluating the effects of inclusion or exclusion of specific instruments on the sensitivity of the radar plots in measuring a successful treatment outcome.

The work presented here outlines a framework for this development, which can be used for tinnitus and for other chronic diseases. One important consideration for the optimal use of this visualization method is the selection of measurement instruments. For example, single-item visual analog or numeric rating scales tend to be viewed as inferior because (i) they are more vulnerable to random measurement errors, which are more likely to be eliminated with multiple items, (ii) the reliability statistic “internal consistency” cannot be computed, and (iii) they are more vulnerable to unknown biases in meaning and interpretation. Here, our selection was in part pragmatic since it was constrained by the data that were available in the STOP study and in the TRI database. The present methodology is generic in a sense that it can be adapted to include any measurement scales. Another important leverage will be the selection of instruments that measure unique and independent components of a complex health-care condition since this should maximize discriminability between patient profiles. The selection of the instruments is thus an important influencer of the radar plots’ sensitivity to change. The choice of instruments is normally guided by the personal preference of the physician or investigator. However, future synthesis of data originating from multiple centers will require agreement from those heads and other key opinion leaders to use a common set of instruments; something that is challenging given the broad diversity of instruments in current use (22). In this regard, the COMiT initiative is currently in the process of establishing an international consensus-based recommendation of a minimum set of outcome domains and instruments considered critically important for performing clinical trials (23). Such international programs will help defining the core set of measurement instruments. A challenge emerging from such endeavors is to obtain instruments of equivalent reliability and efficiency in different languages and sensitive to culture context (42).

Whether such aggregated visualization of data using radar plots will prove useful to clinicians for the management of complex health-care conditions (e.g., tinnitus) will have to be tested. A preliminary evaluation of the radar plots shows mitigated opinions. Some physicians view it as extremely appealing and practical tool to picture the global status of a patient (tinnitus distress, fear of tinnitus, sensitivity to noise, emotional burden, and quality of life), while others do not foresee in what way the radar plots will enable an optimization of the treatment selection. We predict such opinions will also vary between countries where culture and average socioeconomic status differ, and also where different health-care systems apply (purely social-based health-care system versus insurance-based, clinical versus private practice). Furthermore, some physicians express concerns of exposing tinnitus patients to the long list of questionnaires such as those used in the STOP cohort, which could reinforce the negative thoughts and feelings about tinnitus. The participants in STOP originate from the general population and not from a clinical population, and thus whether such series of questionnaires can used on a clinical group will have to be tested. Future research will also have to evaluate the physician perspective in introducing multiple measures in the assessment of tinnitus burden and provide solid conclusions on the usefulness of such integrated measures in the management and treatment of tinnitus.

We personally view that with the increasing pressure over social health-care systems and the dissatisfaction of patients with regards to the quality of the care, novel methodologies are needed to assess patients at baseline and monitor individual response to a treatment. With the increasing development of value-based health care (43), and the distant monitoring of health conditions, smartphone applications could become routinely used in clinics to monitor patients at distance. In the context of tinnitus, the development of such distant monitoring has been recently initiated by Schlee et al. (44–46). This mobile platform could represent an ideal setting to collect patient data at different follow-up times, whereas the physician frontend would display an automated radar plot of the patient’s status. Our hope is that such a tool would allow an immediate monitoring of treatment progress over time and enable the physician to rapidly readjust a treatment prescription depending on the responsiveness of a patient. Furthermore, the inclusion of adverse events and side effects would help to rapidly terminate an intervention and address appropriate care. A better recognition of a patient status and its comorbidities will likely improve the priorities and treatment prescription. Overall, this work contributes to novel strategies for high quality care of chronic tinnitus patients and its implementation in the general clinic.

## Ethics Statement

The STOP project was approved by the local ethics committee “Regionala etikprövningsnämnden” in Stockholm (#2014/1998-31/4). Collection of data for the Tinnitus Research Initiative database was approved by the Ethics Committee of the University of Regensburg, Germany (#08/046).

## Author Contributions

WS, CC, BL, and BC designed the study. NE collected, extracted, and processed the STOP data. WS and CC analyzed the data. DH helped to develop the scientific arguments and contributed to data interpretation. All authors played a role in writing the manuscript and approved the final version.

## Conflict of Interest Statement

The authors declare that the research was conducted in the absence of any commercial or financial relationships that could be construed as a potential conflict of interest.

## Footnotes

^1^http://stop.ki.se.

^2^www.tinnitus-database.de.
