# Supplementary material for: Visualization of Global Disease Burden for the Optimization of Patient Management and Treatment
Source: Front Med (Lausanne). 2017 Jun 19;4:86. doi: 10.3389/fmed.2017.00086 (PMC5475418; doi:10.3389/fmed.2017.00086)
Supplement: Supplementary file 1 [file Table_1.docx]

**Supplementary table 1**

|  |  |  |  |  |  |  | Constant Tinnitus | | | | | |
| --- | --- | --- | --- | --- | --- | --- | --- | --- | --- | --- | --- | --- |
|  | **Control** | | **Occasional Tinnitus** | | **Constant Tinnitus** | | **THI 0-16** | | **THI 18-56** | | **THI >58** | |
|  | n | % | n | % | n | % | n | % | n | % | n | % |
| **Total group** | 1223 | 51,13% | 547 | 22,87% | 622 | 26,00% | 272 | 43,73% | 305 | 49,04% | 45 | 7,23% |
| ***Sex*** | | | | | | | | | | | | |
| **Men** | 425 | 34,75% | 215 | 39,31% | 357 | 57,40% | 183 | 67,28% | 156 | 51,15% | 18 | 40,00% |
| **Women** | 798 | 65,25% | 332 | 60,69% | 265 | 42,60% | 89 | 32,72% | 149 | 48,85% | 27 | 60,00% |
|  | | | | | | | | | | | | |
| ***Age Group*** | | | | | | | | | | | | |
| **<24** | 18 | 1,48% | 15 | 2,75% | 7 | 1,13% | 2 | 0,74% | 5 | 1,65% | 0 | 0,00% |
| **25-34** | 274 | 22,46% | 124 | 22,71% | 107 | 17,26% | 47 | 17,28% | 52 | 17,16% | 8 | 17,78% |
| **35-44** | 349 | 28,61% | 127 | 23,26% | 129 | 20,81% | 67 | 24,63% | 56 | 18,48% | 6 | 13,33% |
| **45-54** | 310 | 25,41% | 142 | 26,01% | 161 | 25,97% | 62 | 22,79% | 85 | 28,05% | 14 | 31,11% |
| **55-64** | 147 | 12,05% | 75 | 13,74% | 84 | 13,55% | 28 | 10,29% | 47 | 15,51% | 9 | 20,00% |
| **65-74** | 109 | 8,93% | 58 | 10,62% | 107 | 17,26% | 53 | 19,49% | 47 | 15,51% | 7 | 15,56% |
| **75-84** | 13 | 1,07% | 5 | 0,92% | 22 | 3,55% | 12 | 4,41% | 9 | 2,97% | 1 | 2,22% |
| **>85** | 0 | 0,00% | 0 | 0,00% | 3 | 0,48% | 1 | 0,37% | 2 | 0,66% | 0 | 0,00% |
|  | | | | | | | | | | | | |
| ***Marital Status*** | | | | | | | | | | | | |
| **Married** | 543 | 44,40% | 227 | 41,50% | 280 | 45,02% | 123 | 45,22% | 139 | 45,57% | 18 | 40,00% |
| **Living with partner** | 382 | 31,23% | 174 | 31,81% | 161 | 25,88% | 77 | 28,31% | 73 | 23,93% | 11 | 24,44% |
| **Single** | 211 | 17,25% | 101 | 18,46% | 115 | 18,49% | 42 | 15,44% | 64 | 20,98% | 9 | 20,00% |
| **Widow/er** | 14 | 1,14% | 3 | 0,55% | 11 | 1,77% | 4 | 1,47% | 6 | 1,97% | 1 | 2,22% |
| **Divorced** | 73 | 5,97% | 42 | 7,68% | 55 | 8,84% | 26 | 9,56% | 23 | 7,54% | 6 | 13,33% |
| ***Education*** | | | | | | | | | | | | |
| **Middle School** | 13 | 1,06% | 6 | 1,10% | 31 | 4,98% | 7 | 2,57% | 18 | 5,90% | 6 | 13,33% |
| **High School** | 206 | 16,84% | 98 | 17,92% | 134 | 21,54% | 53 | 19,49% | 65 | 21,31% | 16 | 35,56% |
| **University** | 910 | 74,41% | 406 | 74,22% | 375 | 60,29% | 174 | 63,97% | 183 | 60,00% | 18 | 40,00% |
| **Other** | 92 | 7,52% | 37 | 6,76% | 82 | 13,18% | 38 | 13,97% | 39 | 12,79% | 5 | 11,11% |
| **Don't know** | 2 | 0,16% | 0 | 0,00% | 0 | 0,00% | 0 | 0,00% | 0 | 0,00% | 0 | 0,00% |
| ***Yearly income (kSEK)*** | | | | | | | | | | | | |
| **<200** | 107 | 8,75% | 67 | 12,25% | 86 | 13,83% | 30 | 11,03% | 47 | 15,41% | 9 | 20,00% |
| **200 - 450** | 596 | 48,73% | 271 | 49,54% | 308 | 49,52% | 135 | 49,63% | 149 | 48,85% | 24 | 53,33% |
| **>450** | 486 | 39,74% | 188 | 34,37% | 198 | 31,83% | 99 | 36,40% | 91 | 29,84% | 8 | 17,78% |
| **Don't know** | 34 | 2,78% | 21 | 3,84% | 30 | 4,82% | 8 | 2,94% | 18 | 5,90% | 4 | 8,89% |
| ***Employment Status*** | | | | | | | | | | | | |
| **Employed** | 845 | 69,09% | 354 | 64,72% | 351 | 56,43% | 162 | 59,56% | 167 | 54,75% | 22 | 48,89% |
| **Unemployed** | 14 | 1,14% | 7 | 1,28% | 5 | 0,80% | 1 | 0,37% | 3 | 0,98% | 1 | 2,22% |
| **Own business** | 115 | 9,40% | 71 | 12,98% | 79 | 12,70% | 32 | 11,76% | 42 | 13,77% | 5 | 11,11% |
| **Retired** | 111 | 9,08% | 55 | 10,05% | 121 | 19,45% | 60 | 22,06% | 53 | 17,38% | 8 | 17,78% |
| **Sick leave** | 11 | 0,90% | 16 | 2,93% | 30 | 4,82% | 7 | 2,57% | 15 | 4,92% | 8 | 17,78% |
| **Parental leave** | 47 | 3,84% | 9 | 1,65% | 7 | 1,13% | 3 | 1,10% | 4 | 1,31% | 0 | 0,00% |
| **Student** | 53 | 4,33% | 26 | 4,75% | 14 | 2,25% | 4 | 1,47% | 9 | 2,95% | 1 | 2,22% |
| **Sabbatical** | 0 | 0,00% | 1 | 0,18% | 0 | 0,00% | 0 | 0,00% | 0 | 0,00% | 0 | 0,00% |
| **Housewife/husband** | 6 | 0,49% | 1 | 0,18% | 0 | 0,00% | 0 | 0,00% | 0 | 0,00% | 0 | 0,00% |
| **Other** | 20 | 1,64% | 7 | 1,28% | 15 | 2,41% | 3 | 1,10% | 12 | 3,93% | 0 | 0,00% |
| **Don't know** | 1 | 0,08% | 0 | 0,00% | 0 | 0,00% | 0 | 0,00% | 0 | 0,00% | 0 | 0,00% |
| ***Hearing Problem*** | | | | | | | | | | | | |
| **Yes** | 285 | 23,30% | 215 | 39,31% | 353 | 56,75% | 145 | 53,31% | 178 | 58,36% | 30 | 66,67% |
| **No** | 763 | 62,39% | 230 | 42,05% | 180 | 28,94% | 89 | 32,72% | 82 | 26,89% | 9 | 20,00% |
| **Don't know** | 175 | 14,31% | 102 | 18,65% | 89 | 14,31% | 38 | 13,97% | 45 | 14,75% | 6 | 13,33% |
| ***Problem tolerating sound*** | | | | | | | | | | | | |
| **Never** | 102 | 8,34% | 30 | 5,48% | 23 | 3,70% | 15 | 5,51% | 8 | 2,62% | 0 | 0,00% |
| **Rarely** | 360 | 29,44% | 106 | 19,38% | 85 | 13,67% | 63 | 23,16% | 22 | 7,21% | 0 | 0,00% |
| **Sometimes** | 538 | 43,99% | 246 | 44,97% | 234 | 37,62% | 113 | 41,54% | 112 | 36,72% | 9 | 20,00% |
| **Usually** | 174 | 14,23% | 115 | 21,02% | 166 | 26,69% | 55 | 20,22% | 97 | 31,80% | 14 | 31,11% |
| **Always** | 49 | 4,01% | 50 | 9,14% | 114 | 18,33% | 26 | 9,56% | 66 | 21,64% | 22 | 48,89% |
| ***Diagnosis of other diseases*** | | | | | | | | | | | | |
| **Yes** | 318 | 26,00% | 155 | 28,34% | 227 | 36,50% | 97 | 35,66% | 113 | 37,05% | 17 | 37,78% |
| **No** | 905 | 74,00% | 392 | 71,66% | 395 | 63,50% | 175 | 64,34% | 192 | 62,95% | 28 | 62,22% |
| ***Medication*** | | | | | | | | | | | | |
| **Yes** | 428 | 35,00% | 215 | 39,31% | 266 | 42,77% | 106 | 38,97% | 135 | 44,26% | 25 | 55,56% |
| **No** | 795 | 65,00% | 332 | 60,69% | 356 | 57,23% | 166 | 61,03% | 170 | 55,74% | 20 | 44,44% |
