# Supplementary material for: Visualization of Global Disease Burden for the Optimization of Patient Management and Treatment
Source: Front Med (Lausanne). 2017 Jun 19;4:86. doi: 10.3389/fmed.2017.00086 (PMC5475418; doi:10.3389/fmed.2017.00086)
Supplement: Supplementary file 2 [file Table_2.docx]

**Supplementary Table 2**

| **CGI score** | **Mean Pre (95% CI)** | **Mean Post (95% CI)** | ***p* value** | **t** | **df** |
| --- | --- | --- | --- | --- | --- |
| ***Very much better (n = 18)*** | |  |  |  |  |
| THI | 51,11 (38,85-63,37) | 14,22 (4,34-24,1) | ***0,0001 | 4,478 | 374 |
| Tsev | 63,33 (54,23-72,44) | 42,22 (31,76-52,68) | 0,1185 | 2,563 | 374 |
| Tloud | 51,67 (38,2-65,14) | 25,56 (12,47-38,64) | *0,0182 | 3,17 | 374 |
| Tunconf | 54,44 (40,34-68,55) | 21,11 (7,86-34,36) | ***0,0007 | 4,047 | 374 |
| Tann | 56,67 (41,97-71,36) | 19,44 (6,05-32,84) | ***< 0,0001 | 4,519 | 374 |
| Tign | 51,11 (36,51-65,71) | 18,33 (7,44-29,23) | ***0,0009 | 3,979 | 374 |
| Tunp | 52,78 (38,01-67,54) | 21,11 (7,49-34,73) | ***0,0016 | 3,844 | 374 |
| Qph | 31,19 (22,54-39,84) | 29,42 (20,48-38,36) | > 0,9999 | 0,2152 | 374 |
| Qps | 36,48 (28,34-44,62) | 27,22 (19,04-35,41) | > 0,9999 | 1,124 | 374 |
| Qso | 30,74 (23,88-37,6) | 27,04 (17,68-36,39) | > 0,9999 | 0,4496 | 374 |
| Qen | 32,64 (25,02-40,25) | 26,01 (16,58-35,44) | > 0,9999 | 0,8045 | 374 |
|  |  |  |  |  |  |
| ***Much better (n = 66)*** | |  |  |  |  |
| THI | 46,76 (41,27-52,25) | 28,45 (23,2-33,71) | ***< 0,0001 | 5,321 | 1430 |
| Tsev | 66,97 (62,45-71,49) | 52,42 (48,96-55,89) | ***0,0003 | 4,228 | 1430 |
| Tloud | 62,12 (56,64-67,6) | 43,94 (39,73-48,15) | ***< 0,0001 | 5,285 | 1430 |
| Tunconf | 67,88 (62,13-73,63) | 47,88 (42,81-52,95) | ***< 0,0001 | 5,814 | 1430 |
| Tann | 63,79 (57,25-70,33) | 44,09 (37,76-50,42) | ***< 0,0001 | 5,726 | 1430 |
| Tign | 61,82 (55,69-67,95) | 43,64 (38,67-48,6) | ***< 0,0001 | 5,285 | 1430 |
| Tunp | 65,15 (58,83-71,47) | 44,24 (38,79-49,7) | ***< 0,0001 | 6,078 | 1430 |
| Qph | 29,87 (26,44-33,3) | 25,48 (22,07-28,88) | > 0,9999 | 1,277 | 1430 |
| Qps | 30,35 (27,23-33,48) | 27,26 (24,25-30,27) | > 0,9999 | 0,8985 | 1430 |
| Qso | 26,11 (22,46-29,77) | 24,29 (21,18-27,4) | > 0,9999 | 0,5285 | 1430 |
| Qen | 27,3 (23,85-30,75) | 24,25 (20,88-27,63) | > 0,9999 | 0,8862 | 1430 |
|  |  |  |  |  |  |
| ***Minimally better (n = 131)*** | |  |  |  |  |
| THI | 49,74 (46,08-53,4) | 37,57 (33,75-41,39) | ***< 0,0001 | 5,208 | 2860 |
| Tsev | 70,23 (67,56-72,89) | 60 (57,63-62,37) | ***0,0001 | 4,378 | 2860 |
| Tloud | 64,2 (60,77-67,63) | 53,05 (49,53-56,58) | ***< 0,0001 | 4,77 | 2860 |
| Tunconf | 72,82 (69,45-76,2) | 57,86 (53,7-62,02) | ***< 0,0001 | 6,404 | 2860 |
| Tann | 68,78 (64,65-72,9) | 54,2 (49,67-58,73) | ***< 0,0001 | 6,241 | 2860 |
| Tign | 66,64 (63,04-70,24) | 54,27 (50,42-58,13) | ***< 0,0001 | 5,293 | 2860 |
| Tunp | 68,78 (65,14-72,42) | 54,35 (50,56-58,14) | ***< 0,0001 | 6,175 | 2860 |
| Qph | 28,7 (26,17-31,23) | 25,87 (23,58-28,17) | > 0,9999 | 1,209 | 2860 |
| Qps | 30,45 (28,21-32,7) | 26,98 (24,83-29,13) | > 0,9999 | 1,486 | 2860 |
| Qso | 27,63 (24,98-30,28) | 26,44 (23,66-29,22) | > 0,9999 | 0,5119 | 2860 |
| Qen | 21,09 (19,09-23,09) | 19,74 (17,74-21,74) | > 0,9999 | 0,5788 | 2860 |
|  |  |  |  |  |  |
| ***No change (n = 255)*** | | | | | |
| THI | 46,23 (43,56-48,89) | 43,07 (40,15-46) | 0,6385 | 1,896 | 5588 |
| Tsev | 70,12 (68,18-72,05) | 67,84 (65,93-69,76) | > 0,9999 | 1,368 | 5588 |
| Tloud | 67,61 (65,11-70,11) | 67,22 (64,75-69,68) | > 0,9999 | 0,2358 | 5588 |
| Tunconf | 73,69 (71,29-76,08) | 70,9 (68,41-73,39) | > 0,9999 | 1,674 | 5588 |
| Tann | 71,22 (68,25-74,18) | 69,18 (66,22-72,13) | > 0,9999 | 1,226 | 5588 |
| Tign | 69,41 (66,78-72,04) | 67,25 (64,63-69,88) | > 0,9999 | 1,297 | 5588 |
| Tunp | 69,69 (67,03-72,35) | 67,61 (64,87-70,35) | > 0,9999 | 1,25 | 5588 |
| Qph | 28,44 (26,54-30,34) | 28,46 (26,61-30,32) | > 0,9999 | 0,0134 | 5588 |
| Qps | 30,64 (29-32,28) | 30,1 (28,48-31,72) | > 0,9999 | 0,3254 | 5588 |
| Qso | 26,97 (25,19-28,74) | 27,1 (25,24-28,95) | > 0,9999 | 0,0786 | 5588 |
| Qen | 21,08 (19,53-22,64) | 20,79 (19,28-22,3) | > 0,9999 | 0,1774 | 5588 |
|  |  |  |  |  |  |
| ***Minimally worse (n = 77)*** | |  |  |  |  |
| THI | 50,81 (45,75-55,86) | 53,35 (48,15-58,55) | > 0,9999 | 0,9082 | 1672 |
| Tsev | 70,91 (67,39-74,43) | 78,18 (75,09-81,27) | 0,105 | 2,595 | 1672 |
| Tloud | 67,53 (63,32-71,74) | 76,1 (72,25-79,96) | *0,0249 | 3,058 | 1672 |
| Tunconf | 74,03 (69,75-78,3) | 80 (76,45-83,55) | 0,3651 | 2,132 | 1672 |
| Tann | 68,7 (63,17-74,24) | 79,22 (74,82-83,63) | **0,002 | 3,753 | 1672 |
| Tign | 68,96 (64,13-73,79) | 77,01 (72,93-81,1) | *0,0453 | 2,873 | 1672 |
| Tunp | 68,96 (64,31-73,61) | 78,96 (75,27-82,65) | **0,0041 | 3,568 | 1672 |
| Qph | 28,15 (25,14-31,16) | 30,09 (27,17-33) | > 0,9999 | 0,6907 | 1672 |
| Qps | 29,7 (26,76-32,64) | 31,04 (28,15-33,93) | > 0,9999 | 0,4788 | 1672 |
| Qso | 26,23 (22,64-29,83) | 27,01 (23,45-30,58) | > 0,9999 | 0,278 | 1672 |
| Qen | 18,86 (16,81-20,91) | 19,73 (17,66-21,8) | > 0,9999 | 0,3095 | 1672 |
|  |  |  |  |  |  |
| ***Much worse (n = 24)*** | |  |  |  |  |
| THI | 45,58 (36,48-54,68) | 55,33 (44,9-65,77) | 0,5251 | 1,985 | 506 |
| Tsev | 74,17 (66,53-81,8) | 80,83 (74,38-87,29) | > 0,9999 | 1,357 | 506 |
| Tloud | 74,17 (67,82-80,51) | 84,17 (79,77-88,57) | 0,4657 | 2,035 | 506 |
| Tunconf | 75,83 (67,84-83,83) | 84,58 (79,05-90,11) | 0,8307 | 1,781 | 506 |
| Tann | 73,33 (63,28-83,39) | 87,08 (81,62-92,55) | 0,0586 | 2,799 | 506 |
| Tign | 72,08 (65,11-79,06) | 83,33 (77,36-89,31) | 0,2469 | 2,29 | 506 |
| Tunp | 72,5 (64,74-80,26) | 85,83 (81,59-90,07) | 0,0757 | 2,714 | 506 |
| Qph | 28,71 (22,05-35,37) | 33,33 (26,59-40,07) | > 0,9999 | 0,941 | 506 |
| Qps | 28,33 (22,81-33,85) | 33,19 (26,52-39,87) | > 0,9999 | 0,9894 | 506 |
| Qso | 21,67 (15,93-27,41) | 25 (19,05-30,95) | > 0,9999 | 0,6785 | 506 |
| Qen | 17,4 (12,66-22,13) | 18,65 (13,65-23,64) | > 0,9999 | 0,2544 | 506 |
|  |  |  |  |  |  |
